# New insight into magneto-structural phase transitions in layered TbMn_2_Ge_2_-based compounds

**DOI:** 10.1038/srep45814

**Published:** 2017-04-04

**Authors:** Chunsheng Fang, Guoxing Li, Jianli Wang, W. D. Hutchison, Q. Y. Ren, Zhenyan Deng, Guohong Ma, Shixue Dou, S. J. Campbell, Zhenxiang Cheng

**Affiliations:** 1 Institute for Superconducting and Electronic Materials, Innovation Campus, University of Wollongong, NSW 2500, Australia; 2 State Key Laboratory on Integrated Optoelectronics, College of Electronic Science and Engineering, Jilin University, Changchun 130012, People’s Republic of China; 3 College of Physics, Jilin University, Changchun 130012, People’s Republic of China; 4 School of Physical, Environmental and Mathematical Sciences, UNSW Canberra at the Australian Defence Force Academy, ACT 2600, Australia; 5 Department of Physics, Shanghai University, 99 Shangda Road, Shanghai, 200444, China

## Abstract

The Tb_1−x_Y_x_Mn_2_Ge_2_ series (x = 0, 0.1, 0.2) compounds are found to exhibit two magnetic phase transitions with decreasing temperature: from the paramagnetic state to the antiferromagnetic interlayer state at T_N_^inter^ and from an antiferromagnetic interlayer structure to a collinear ferrimagnetic interlayer structure at T_C_^inter^. Compared with the slight change of T_N_^inter^ (409 K, 410 K and 417 K for x = 0, 0.1 and 0.2 respectively), the replacement of Y for Tb leads to a significant decrease in T_C_^inter^ from 97.5 K for x = 0 to 74.6 K for x = 0.2. The variation in T_C_^inter^ can be ascribed to the combination of two effects: (1) chemical pressure and (2) magnetic dilution effect by Y substitution for Tb. Besides, a strong anisotropic magnet-volume effect has been detected around T_C_^inter^ in all compounds with Δa/a = 0.125%, 0.124% and 0.130% for x = 0, 0.1 and 0.2, respectively while no obvious effect is detected along the c-axis. The maximum magnetic entropy change were found to be −ΔS_*max*_ = 9.1 J kg^−1^ K^−1^, 11.9 J kg^−1^ K^−1^ and 6.3 J kg^−1^ K^−1^ with a field change from 0 T to 5 T for x = 0, 0.1, 0.2 respectively.

Since the discovery in 1997 of a giant magnetocaloric effect (GMCE) originating from a discontinuous first order magnetic transition in Gd_5_Si_2_Ge_2_^1^, room-temperature magnetic refrigeration based on the magnetocaloric effect (MCE) has attracted significant attention due to its energy efficiency and environment friendly in comparison with conventional gas compression-expansion refrigeration^2^. A number of materials which exhibit giant magnetic entropy changes at magnetic transitions have been investigated, including MnFeP_0.45_As_0.55_^3^, MnAs_1−x_Sb_x_^4^, Ni-Mn-Sn-based alloys^5^, Ni-Mn-Ga^6^,^7^, and La(Fe,Si)_13_^8^. The key features of these systems are the temperature- and magnetic field-induced first-order magneto-structural or magneto-elastic phase transitions. Given these promising developments, magnetic materials which exhibit a large magnetocaloric effect have been studied extensively, both experimentally and theoretically, over the past two decades with the overall aim of increasing the efficiency of magnetic refrigeration techniques^9^,^10^. While a key focus is exploration of materials that exhibit a pronounced magnetocaloric effect at room temperature, materials that operate in the low temperature region are also useful in meeting the cooling requirements for fields such as gas liquefaction or attaining millikelvin for experimental research facilities. However, so far only a few materials such as GdLiF_4_, GdF_3_ and Gd_3_Ga_5_O_12_ are used commercially^11^. As reflected by the increase in exploration of materials which exhibit a large MCE below room temperature^10^,^11^,^12^,^13^, the search for materials which exhibit large magnetocaloric effects over temperature ranges relevant for hydrogen and natural gas liquefaction are also important for exploring potential applications.

Some RT_2_X_2_ compounds (R = rare earth, T = transition metal, and X = Si or Ge) have been found to exhibit large MCE values with small hysteresis losses near their low magnetic transition temperatures^11^,^13^,^14^,^15^,^16^. For example, the magnetic entropy values of RNi_2_Si_2_ (R = Dy, Ho, Er) compounds are 21.3 J kg^−1^ K^−1^, 21.7 J kg^−1^ K^−1^ and 22.9 J kg^−1^ K^−1^ around 6.5 K, 4.5 K and 3.5 K respectively during a change of magnetic induction intensity from 0–5 T^16^, while the magnetic entropy of ErCr_2_Si_2_ attained 29.7 J kg^−1^ K^−1^ near the magnetic ordering temperature 4.5 K^17^. The crystal structure of the RT_2_X_2_ series is body centred tetragonal ThCr_2_Si_2_-type (with space group I4/mmm)^15^,^18^,^19^, with the sequence -R-X-T-X-R- atomic layers stacked along the c-axis. The rare earth elements typically exhibit large magnetic moment (for example μ_Tb_ = 8.8 μ_B_ in TbMn_2_Si_2_ at 5 K)^20^ and correspondingly make a large contribution to the magnetocaloric effect^14^,^15^,^17^. Given the sensitivity of the magnetic state in RMn_2_X_2_ to the intra-planar Mn-Mn spacing^15^,^19^,^21^,^22^,^23^,^24^, compounds in this series are found to display a rich variety of interesting phenomena, including superconductivity, magnetism, mixed valence, heavy fermions, and Kondo behaviour^25^,^26^,^27^. This diversity enables control of the interplay between the R-Mn and Mn-Mn exchange interactions in RMn_2_X_2_ through external factors such as pressure^28^, temperature and magnetic field^29^ meaning that such compounds have the potential for competitive performance^15^,^19^,^24^. The notations used in this paper to describe the magnetic structure type and critical transition temperatures are defined by Venturini *et al*.^22^ Using standard magnetic methods^19^,^30^, TbMn_2_Ge_2_ was reported to be antiferromagnetic below Néel temperature T_N_ = 410 K with the AFil antiferromagnetic interlayer structure (*i.e.* a collinear antiferromagnetic structure between adjacent Mn planes in a + − + − sequence along the *c*-axis^22^.) Below T_C_ = 100 K, TbMn_2_Ge_2_ exhibits a collinear ferrimagnetic structure in which the Tb moments order ferromagnetically and couple antiferromagnetically with the Mn moment^23^. Furthermore, in a later study for the Tb_1−x_Y_x_Mn_2_Ge_2_ series (x = 0–0.4), it was reported that the replacement of Y for Tb leads to significant modifications of both the Curie temperature (from 76 K for TbMn_2_Ge_2_ to almost 0 K for Tb_0.4_Y_0.6_Mn_2_Ge_2_) and magnetovolume effect (the volume effect is ∆V/V = 3.2 × 10^–3^ and 2.7 × 10^−3^ for x = 0 and 0.1 respectively)^30^. The magnetic phase transitions around T_C_ in the Tb-rich Tb_1−x_Y_x_Mn_2_Ge_2_ compounds were shown to be first order^30^, offering scope for large magnetocaloric effects around the region of their Curie temperatures.

Here we present a systematic study of the magnetic transition from antiferromagnetism to ferromagnetism in a series of Tb_1−x_Y_x_Mn_2_Ge_2_ samples (x = 0, 0.1, 0.2) using a combination of methods including variable temperature x-ray diffraction (XRD), specific heat, differential scanning calorimetry (DSC) and magnetization measurements. The overall aim is to understand fully the influence of Y substitution for Tb on magnetocaloric effects and search for novel magnetocaloric materials that may be suitable for operation over the hydrogen and natural gas liquefaction temperature ranges.

## Method

The polycrystalline Tb_1−x_Y_x_Mn_2_Ge_2_ samples with x = 0, 0.1, 0.2 were prepared by arc melting constituent elements of 99.9% purity under argon atmosphere. For improved crystallization and chemical homogeneity, the samples were annealed in vacuum-sealed quartz tube at 850 °C for 7 days after arc melting. The dc magnetic measurements were performed using a Quantum Design 9 T physical properties measurement system (PPMS). The magnetic behaviour was investigated over the range from 5 K to 340 K in a magnetic field 0.01 T. Differential scanning calorimetry measurements were performed on differential scanning calorimetry equipment (DSC 204 ***F1** Phoenix*^*®*^) from 340 K to 500 K. Magnetization-field loops were obtained at temperatures close to the Curie temperature of samples with magnetic fields over the range 0–5 T. The heat capacity measurements were performed on a Quantum Design 14 T physical properties measurement system scanning from 2 K to 250 K. The samples were characterized and the structures determined by variable temperatures XRD measurements over the temperature range (12–300 K) using a PANAlytical diffractometer with Cu-Kα radiation.

## Results and Discussion

### Structural behaviour

**Figure 1:**
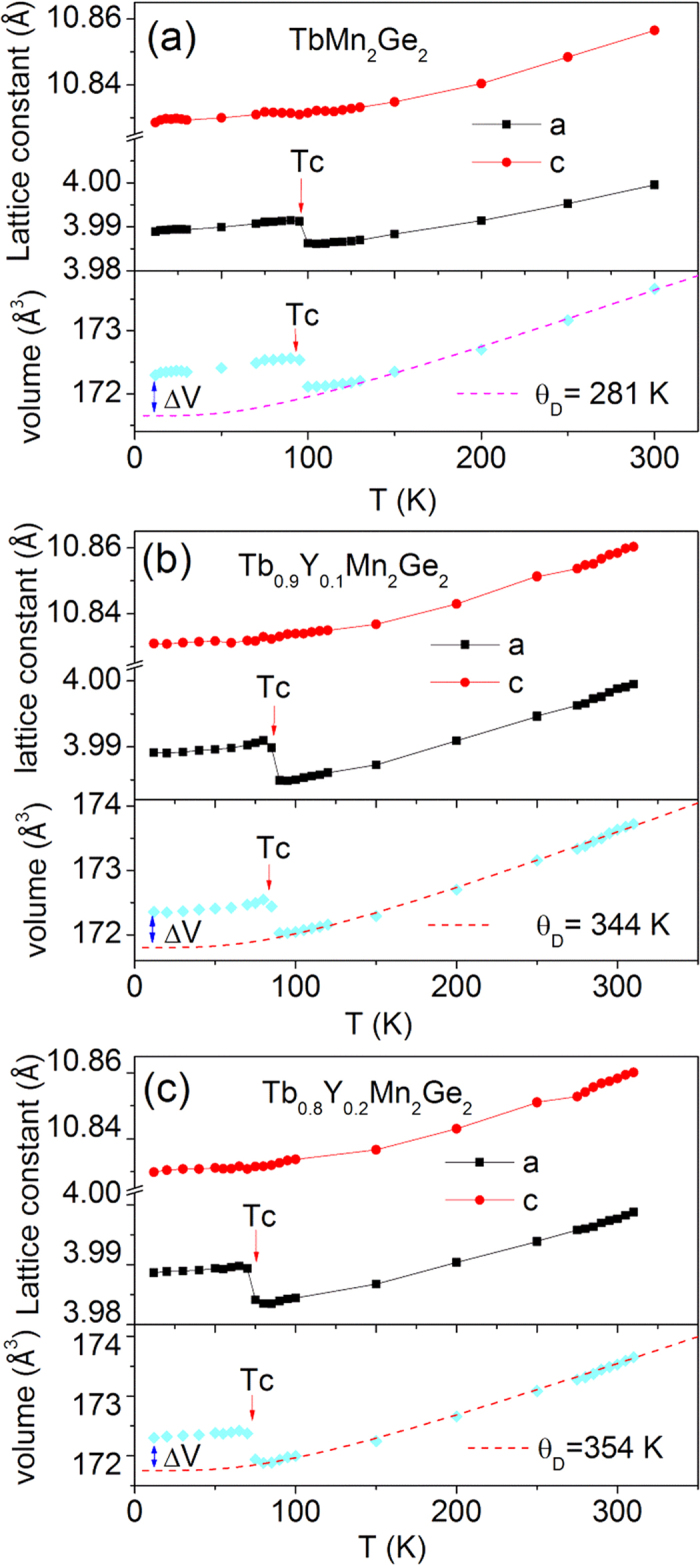
Temperature dependence of the lattice constants a, c and unit cell volume: (**a**) TbMn_2_Ge_2_, (**b**) Tb_0.9_Y_0.1_Mn_2_Ge_2_ and (**c**) Tb_0.8_Y_0.2_Mn_2_Ge_2_. The dashed lines show the phonon contribution to the lattice expansion as evaluated from the Gruneisen relation.

The room temperature x-ray diffraction study shows that all samples are single phase and that patterns can be indexed with a space group of I4/mmm as expected. The Rietveld refinements have been carried out using the FullProf package^31^ with the main results shown in Fig. 1(a,b and c) for x = 0, 0.1 and 0.2 respectively. It can be seen from Fig. 1. that the variations of lattice parameters of *a* and *c* with temperature display strong anisotropy: the lattice parameter *c* (red solid circle) increases monotonically with increasing temperature while a pronounced discontinuity is observed in the *a* lattice parameter (black solid square) around the Curie temperature T_c_ for each sample (the transition temperatures were determined as the point where the value of dM/dT is minimum). Similar behaviours for TbMn_2_Ge_2_ were also determined by Morellon *et al*.^23^ for which an anomaly in the thermal expansion along the *a*-axis was found near T_c_. The discontinuity in the *a* lattice parameter around T_c_ leads to the associated decrease in the unit cell volume for all samples as also evident in Fig. 1. These behaviours are very similar to the behaviour reported for Pr_0.5_Y_0.5_Mn_2_Ge_2_^15^ (i.e. PrMn_2_Ge_2_ diluted by Y), but different from NdMn_2_Ge_0.4_Si_1.6_^32^, NdMn_1.9_Ti_0.1_Si_2_^33^ and NdMn_1.7_Cr_0.3_Si_2_^34^,^35^ (where Mn diluted with transition metal Ti or Ge diluted by Si) for which the lattice parameter *a* decreases with increasing temperature around T_C_ while the lattice parameter *c* expands. In order to derive the magneto-volume effect below T_C_, we have calculated the contribution from lattice vibration using the Debye model:


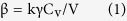


where β is the volume thermal expansion coefficient of the parameter state, k is the compressibility, γ is the Gruneisen constant and C_v_ is the specific heat at constant volume caused by lattice vibrations. C_v_ was derived from the Debye theory of the specific heat using the value of the Debye temperature ^θ^_D_ (as derived from our specific heat measurements for each of the samples as described below for the three compositions):


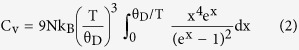


where k_B_ is the Boltzmann constant and N is the number of the atoms. The thermal expansion for the hypothetical paramagnetic state is derived on integrating Eq. (2) with respect to temperature. The parameter 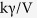 was adjusted to obtain the best least-squares fitting to the successive data points of the observed thermal expansion curve well above the magnetic ordering temperature (based on the fact that the magnetic contribution in the antiferromagnetic region to total thermal expansion can be ignored for these types of compounds)^32^.

The temperature dependence of the unit cell volumes based on Debye theory for the TbMn_2_Ge_2_, Tb_0.9_Y_0.1_Mn_2_Ge_2_ and Tb_0.8_Y_0.2_Mn_2_Ge_2_ samples are shown by the dashed lines in Fig. 1(a,b and c) with pronounced magneto-volume effects evident below their magnetic transition temperatures T_C_ = 94 K, T_C_ = 83 K and T_C_ = 70 K respectively. The discontinuous nature of the changes in *a* lattice parameter and unit cell volume *V* at the Curie temperatures as shown in Fig. 1, are consistent with the first order nature of these transitions as discussed fully below. The changes in the lattice parameter *a* are Δa/a = 0.125%, Δa/a = 0.124% and Δa/a = 0.130% for x = 0, 0.1 and 0.2 respectively with spontaneous volume magnetostriction ω_s_ (=ΔV_m_/V) at 5 K determined as: TbMn_2_Ge_2_ - ω_s_ = 4.1 × 10^−3^; Tb_0.9_Y_0.1_Mn_2_Ge_2_ - ω_s_ = 3.2 × 10^−3^ and Tb_0.8_Y_0.2_Mn_2_Ge_2_ - ω_s_ = 5.8 × 10^−3^.

### Magnetic phase transition

**Figure 2:**
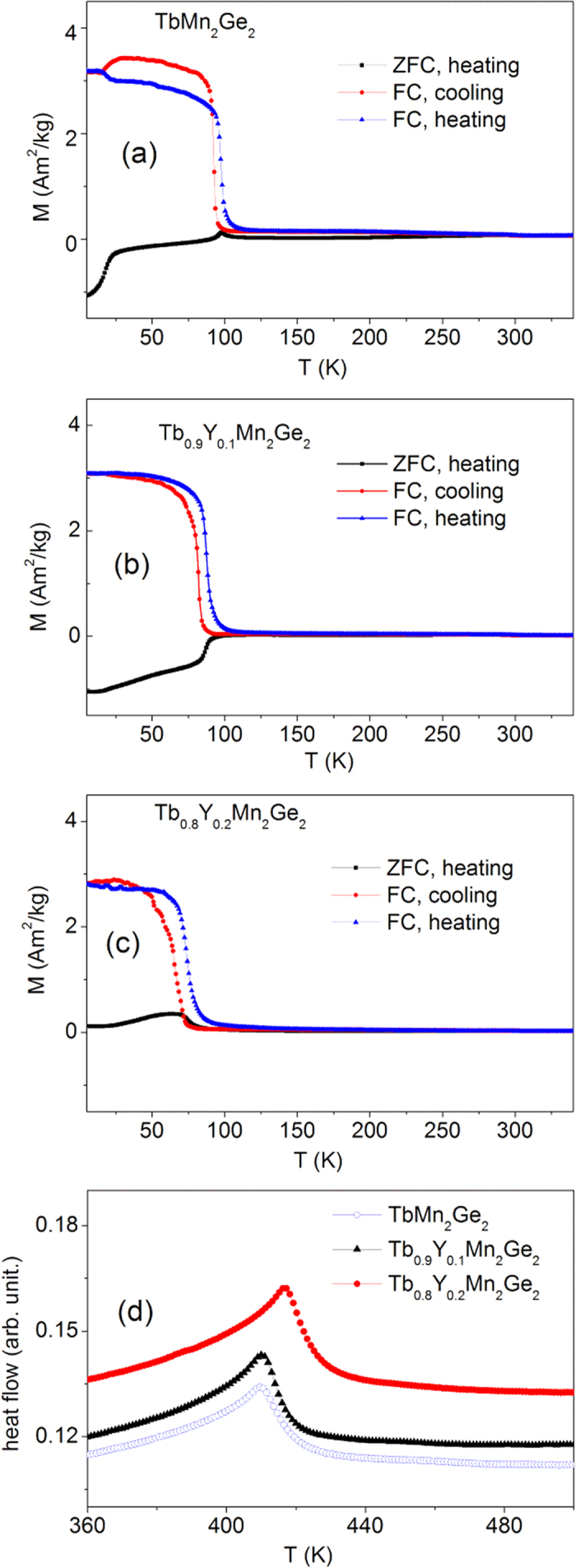
Temperature dependence of magnetization on ZFC heating, FC cooling and FC heating processes under a field of B = 0.01 T: (**a**) TbMn_2_Ge_2_, (**b**) Tb_0.9_Y_0.1_Mn_2_Ge_2_ and (**c**) Tb_0.8_Y_0.2_Mn_2_Ge_2_. (**d**) the differential scanning calorimetry curves for the three samples over the range ~300–500 K.

The magnetisation of the three samples have been measured in a field of B = 0.01 T over the temperature range 5–340 K. As in Fig. 2(a,b and c) the TbMn_2_Ge_2_, Tb_0.9_Y_0.1_Mn_2_Ge_2_ and Tb_0.8_Y_0.2_Mn_2_Ge_2_ samples were respectively measured on warming from 5 K in three states: after cooling in zero field (ZFC heating) and after cooling and heating in a field of B = 0.01 T (FC cooling and FC heating). As is evident from the magnetization versus temperature curves of Fig. 2(a,b and c), there is an abrupt change in magnetisation at the Curie temperature T_C_^inter^ that marks the magnetic phase transition from a collinear antiferromagnetism (AFil)^22^ at higher temperature to a collinear ferrimagnetic structure along the *c* axis at lower temperature according to the neutron diffraction study on TbMn_2_Ge_2_^23^. Of the three samples, TbMn_2_Ge_2_ has the highest T_C_^inter^(warm) = 97.5 K and T_C_^inter^(cool) = 93.0 K transitions respectively as determined from the FC heating and cooling M-T curves, while the values for Tb_0.9_Y_0.1_Mn_2_Ge_2_ are derived to be T_C_^inter^(warm) = 87.5 K and T_C_^inter^(cool) = 81.8 K with the values for Tb_0.8_Y_0.2_Mn_2_Ge_2_ being T_C_^inter^(warm) = 74.6 K and T_C_^inter^ cool) = 66.0 K (normally the transition temperature during the FC process is chosen as the Curie temperature T_c_). As expected, the higher the level of doping of non-magnetic Y atoms in Tb_1−x_Y_x_Mn_2_Ge_2_, the lower the magnetic phase transition temperature^30^.

Differential scanning calorimetry measurements have been carried out on the Tb_1−x_Y_x_Mn_2_Ge_2_ samples over the temperature range 300–500 K (Fig. 2(d)) in order to investigate the paramagnetic to antiferromagnetic transition^10^,^23^ at T_N_^inter^. As revealed by the DSC results in Fig. 2(d), the T_N_^inter^ transition temperatures are found to increase slightly with increasing Y concentration - T_N_^inter^ = 409 K, T_N_^inter^ = 410 K and T_N_^inter^ = 417 K for x = 0.0, 0.1 and 0.2 respectively. Compared with the reduction in ferromagnetic transition temperature on replacement of Tb atoms by Y atoms, the paramagnetic to antiferromagnetic transition temperatures are found to exhibit a slight increase (Fig. 2). The increase in T_N_^inter^ values is due to enhancement of the Mn-Mn exchange interaction as a result of the slight reduction of Mn-Mn distance. This behaviour is similar to the PrMn_2_Ge_2−x_Si_x_ system^36^ in which the paramagnetic to antiferromagnetic transition temperatures are found to increase slightly while the antiferromagnetic to ferromagnetic transition temperatures decrease on replacing Ge with Si.

**Figure 3:**
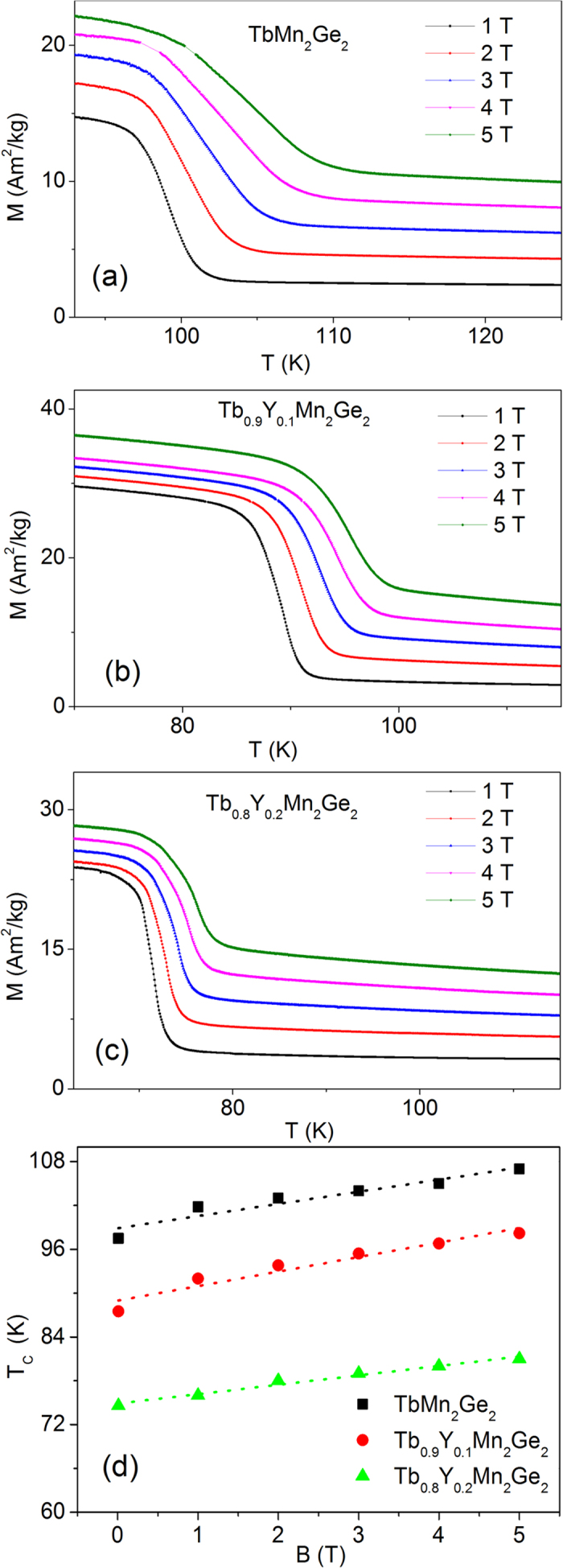
The magnetisation versus temperature curves during cooling under various magnetic field (B = 1–5 T). (**a**) TbMn_2_Ge_2_, (**b**) Tb_0.9_Y_0.1_Mn_2_Ge_2_, (**c**) Tb_0.8_Y_0.2_Mn_2_Ge_2_. (**d**) The variation of ferromagnetic transition temperature T_c_ with magnetic field for the three samples. The dashed lines represent linear fits to the T_c_-B curves leading to dT_c_/dB values for each sample.

The temperature dependences of magnetization for TbMn_2_Ge_2_, Tb_0.9_Y_0.1_Mn_2_Ge_2_ and Tb_0.8_Y_0.2_Mn_2_Ge_2_ under various external magnetic fields are presented in Fig 3(a,b and c) respectively. As expected the ferromagnetic transition temperature T_C_^inter^ is shifted to higher temperature with increase in applied magnetic field. For example, the transition temperatures are T_C_^inter^ = 101.8 K, T_C_^inter^ = 92 K and T_C_^inter^ = 76 K for TbMn_2_Ge_2_, Tb_0.9_Y_0.1_Mn_2_Ge_2_ and Tb_0.8_Y_0.2_Mn_2_Ge_2_ respectively in an external magnetic field of B = 1 T, while the transition temperatures are shifted to T_C_^inter^ = 107 K, T_C_^inter^ = 98.2 K and T_C_^inter^ = 81 K respectively in a field of B = 5 T. The field dependence of the magnetic transition temperatures are summarized in Fig. 3(d). The values of dT_c_/dB (obtained on linear fitting of the experimental data in Fig. 3(d) along with a summary of experimental data determined for Tb_1−x_Y_x_Mn_2_Ge_2_ (x = 0, 0.1, 0.2) in this investigation, are provided in Table 1.

### Y doping in Tb_1−x_Y_x_Mn_2_Ge_2_ - Chemical pressure effect

As noted above, the effect of replacing the magnetic rare earth Tb with the nonmagnetic ion Y in Tb_1−x_Y_x_Mn_2_Ge_2_ is to weaken the exchange interaction between magnetic ions due to the dilution effect. The magnetic behaviour of Y-doped Tb_1−x_Y_x_Mn_2_Ge_2_ will also be modified as a result of chemical pressure due to differences in the atomic radii of the Tb(1.80 Å) and Y(1.78 Å) ions and resultant changes in lattice parameters. In order to separate these two contributions - dilution effect and pressure effect - and their influence on the variation in magnetic transition temperature, the decrease of T_C_ by chemical pressure was calculated as follows. The chemical pressure Δp was calculated^20^,^22^,^33^ according to the Murnaghan equation below:


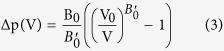


where V_0_, B_0_ and 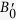 are the volume, the bulk modulus and its first derivative of TbMn_2_Ge_2_ and V is the volume of the unit cell at room temperature of the Y doped samples. Here, due to the similarity of crystal structure for the RMn_2_Ge_2_ system, we assume that the values of B_0_ and 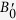 for PrMn_2_Ge_2_ (B_0_ = 38.0 Gpa, 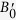 = 19.5 as derived from our synchrotron data under external pressure^37^) can be applied to TbMn_2_Ge_2_ at room temperature. Given that the doped materials Tb_1−x_Y_x_Mn_2_Ge_2_ (x = 0.1, 0.2) retain the ThCr_2_Si_2_-type tetragonal structure, the chemical pressure Δp caused by doping can be assumed to have the same effect as mechanical pressure. According to previous findings that describe the pressure effect on the magnetic properties of TbMn_2_Ge_2_ (dT_c_/dP = −2.9 K/kbar)^38^, the values of ΔT_C_ can be deduced by the relationship:


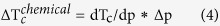


where Δp is the calculated chemical pressure. The calculated values of ΔT_C_ for Tb_0.9_Y_0.1_Mn_2_Ge_2_ and Tb_0.8_Y_0.2_Mn_2_Ge_2_ are ΔT_C = _1.94 K and ΔT_C_ = 8.7 K respectively. As noted above (see also Fig. 1, Figs 2 and 3.) the effect of replacing Tb atoms with Y atoms in Tb_1−x_Y_x_Mn_2_Ge_2_ also contributes to the decrease in the Curie temperature. It can therefore be concluded that chemical pressure accounts for ~17.3% and ~32.2% of the decrease in transition temperatures for x = 0.1 and 0.2 respectively. In addition, the value of dT_c_/dp can be derived using the Clausius-Clapeyron thermodynamic relation as follows:^15^


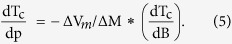


The values of the change in the unit cell volume ΔV_m_ he change in moment Δμ during magnetic phase transition around T_C_ and dT_c_/dB for each sample were taken from the present experimental results listed in Table.1. The derived results are dT/dp = −3.03 K/kbar, −2.84 K/kbar and −3.07 K/kbar for TbMn_2_Ge_2_, Tb_0.9_Y_0.1_Mn_2_Ge_2_ and Tb_0.8_Y_0.2_Mn_2_Ge_2_, respectively. These calculated values are in general accord with the value of dT_c_/dp = −2.9 K/kbar for TbMn_2_Ge_2_^38^, deviating by ~4.5%, ~2.1% and ~5.9% for the x = 0.0. 0.1, 0.2 samples respectively.

### Magnetocaloric effect

**Figure 4:**
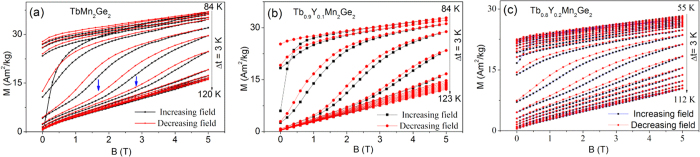
Curves of isothermal magnetization versus magnetic field at temperatures around T_c_. (**a**) TbMn_2_Ge_2_, (**b**) Tb_0.9_Y_0.1_Mn_2_Ge_2_ and (**c**) Tb_0.8_Y_0.2_Mn_2_Ge_2_.

Graphs of the magnetization as a function of applied field are shown for TbMn_2_Ge_2_, Tb_0.9_Y_0.1_Mn_2_Ge_2_, and Tb_0.8_Y_0.2_Mn_2_Ge_2_ at temperatures around T_C_^inter^ in Fig. 4(a,b and c) respectively. It can be seen that with increasing temperature beyond T_C_^inter^, a field-induced metamagnetic phase transition from the antiferromagnetic state to the ferromagnetic state at certain temperatures has been detected. The region of the metamagnetic phase transition for TbMn_2_Ge_2_ is indicated by arrows in Fig. 4(a) as a typical example. This behaviour indicates that the region of ferromagnetic ordering in Tb_1−x_Y_x_Mn_2_Ge_2_ can be shifted to higher temperatures by a stronger applied magnetic field.

**Figure 5:**
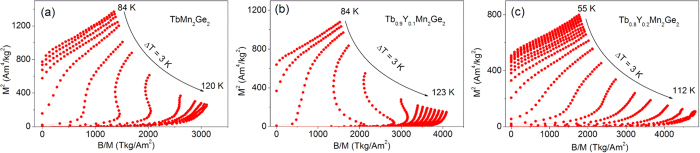
Arrott plots: isotherms graphs of M^2^ versus B/M for decreasing magnetic fields at temperatures around T_c_. (**a**) TbMn_2_Ge_2_, (**b**) Tb_0.9_Y_0.1_Mn_2_Ge_2_ and (**c**) Tb_0.8_Y_0.2_Mn_2_Ge_2_.

The nature of the magnetic transitions (first order or second order) was analysed using Arrott plots with the magnetisation expressed in the usual way as graphs of M^2^ versus B/M (Fig. 5). As can be seen in Fig. 5(a,b and c), negative slopes are detected in the M^2^ versus B/M graphs for the TbMn_2_Ge_2_ and Tb_0.9_Y_0.1_Mn_2_Ge_2_ samples thus indicating that the antiferromagnetic to ferromagnetic processes are first order^39^. However, Some papers^40^,^41^ reported that for compounds near the critical point (from first order to second order magnetic phase transition) such as DyCo_2_, this criterion of Arrott plots do not always work properly. It is also noted that the negative slopes for Tb_0.8_Y_0.2_Mn_2_Ge_2_ around the antiferromagnetic to ferromagnetic transition was reduced compared with those for the TbMn_2_Ge_2_ and Tb_0.9_Y_0.1_Mn_2_Ge_2_ samples. However, the first order transition characters of all the three samples can be confirmed from our variable temperatures crystal structure analyses above, where strong magneto-elastic coupling around T_C_^inter^ has been detected (Fig. 1).

The magnetic entropy changes ΔS_M_ for all samples have been determined from the isothermal magnetization curves of Fig. 4(a,b and c), by using the standard Maxwell relationship:


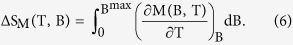


**Figure 6:**
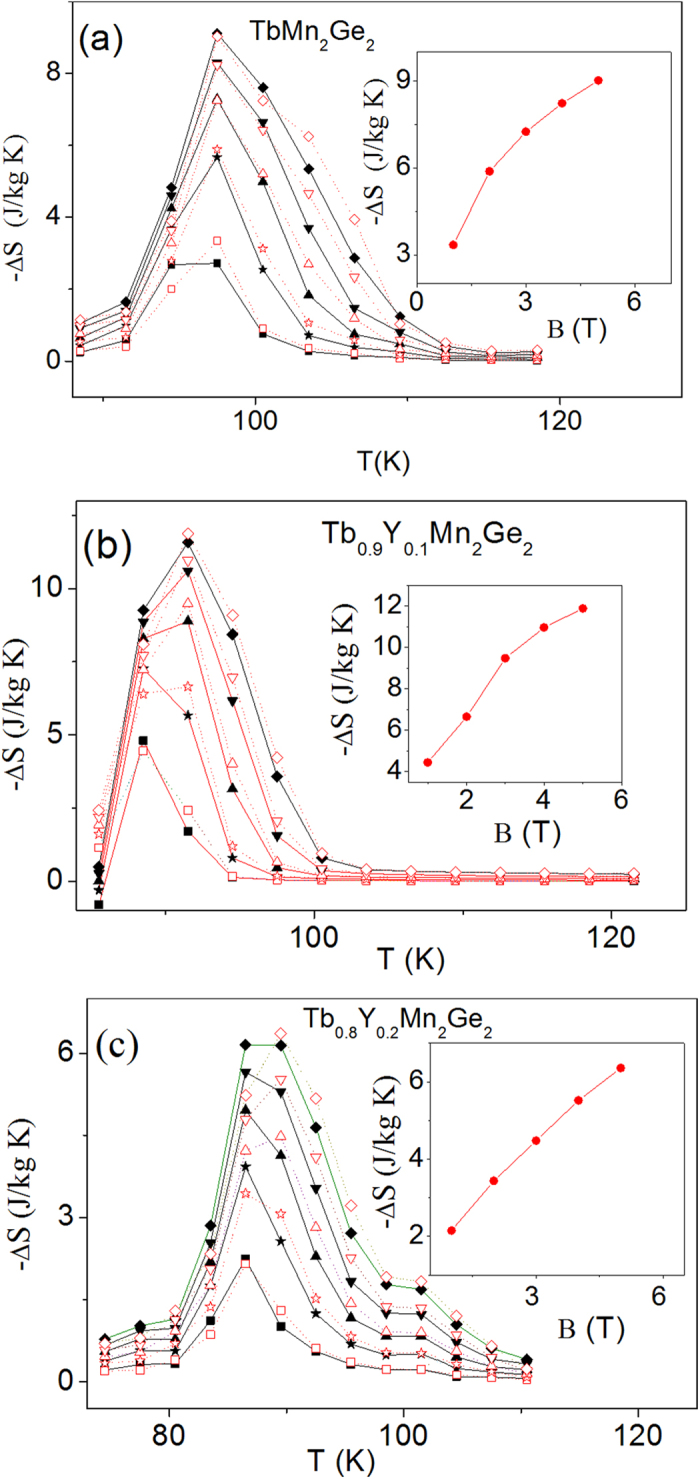
The magnetic entropy changes around the ferromagnetic transition temperatures for applied magnetic fields from 1–5T. (**a**) TbMn_2_Ge_2_, (**b**) Tb_0.9_Y_0.1_Mn_2_Ge_2_ and (**c**) Tb_0.8_Y_0.2_Mn_2_Ge_2_ (black full symbols ◾ ★ ▴ ▾ ♦ for 1–5 T respectively during increasing field and the red empty symbols correspond to 1–5 T for the decreasing field). The insets show the variation of the maximum values of magnetic entropy changes for the decreasing field values.

The calculated temperature dependent magnetic entropy changes for the Tb_1−x_Y_x_Mn_2_Ge_2_ samples with x = 0, 0.1 and 0.2 for both increasing field and decreasing field processes between field changes of ΔB = 0–1 T and ΔB = 0–5 T are shown in Fig. 6(a,b and c) respectively with the maximum values ΔS_*max*_ shown as a function of applied field in the insets of Fig. 6. With a field change of ΔB = 0–5 T, the value of −ΔS_*max*_ are 9.1 J/kgK, 11.9 J/kgK and 6.3 J/kgK for TbMn_2_Ge_2_, Tb_0.9_Y_0.1_Mn_2_Ge_2_ and Tb_0.8_Y_0.2_Mn_2_Ge_2_ respectively, demonstrating that the entropy change for Tb_0.9_Y_0.1_Mn_2_Ge_2_ is the largest of the three samples. As it is clear from Fig. 4, while TbMn_2_Ge_2_ has the highest fraction of magnetic rare earth element and largest saturation magnetization (42.5 Am^2^/kg at 84 K), its large hysteresis loss (7.40 J/kg) leads to reduction in the magnetic entropy change. By comparison, with the lowest concentration of magnetic rare earth Tb, the Tb_0.8_Y_0.2_Mn_2_Ge_2_ sample displays the lowest saturation magnetization (only 32.5 Am^2^/kg even at 55 K) and the smallest hysteresis loss 5.21 J/kg), while as shown in Fig. 4(b), Tb_0.9_Y_0.1_Mn_2_Ge_2_ with medium concentration of Tb has a relatively large saturation magnetization of 38.0 Am^2^/Kg at 84 K and small hysteresis loss (5.36 J/kg). The refrigerant capacity (RCP), defined as the product of −ΔS_*max*_ and the full width at half maximum of the −ΔS_*max*_ curve, for the three samples are: 93.3 J/kg, 102.9 J/kg, 62.4 J/kg for TbMn_2_Ge_2_, Tb_0.9_Y_0.1_Mn_2_Ge_2_ and Tb_0.8_Y_0.2_Mn_2_Ge_2_ respectively, with a field change of ΔB = 0–5 T. The MCE value of Tb_0.9_Y_0.1_Mn_2_Ge_2_ is comparable to those of other materials for a field change of ΔB = 0–5 T including: GdCoAl, −ΔS_max_(T) = 10.4 J/kgK at 100 K^34^, TbCoAl, −ΔS_max_(T) = 10.5 J/kgK at 70 K^34^ and GdMn_2_Ge_2_, −ΔS_max_(T) = 1.2 J/kgK at 95 K^28^, all of which, in common with Tb_0.9_Y_0.1_Mn_2_Ge_2_, importantly exhibit negligible field and thermal hysteresis losses.

Moreover, it is well accepted that first-order phase transitions are accompanied by a latent heat and the barocaloric effect can be expected. In fact giant barocaloric effect has been found in several systems recently including Mn_3_GaN (∆S_bar_ = 22.3 J/K kg)^42^ and Ni–Mn–In magnetic superelastic alloys (∆S_bar_ = 27.7 J/K kg)^43^. Based on the fact that all these three Tb_1−x_Y_x_Mn_2_Ge_2_ samples exhibit strong magnetovolume effect around magnetic phase transition, we have calculated the barocaloric effect using the Clausius_Clapeyron relation^43^. The barocaloric effects entropy change ∆S_bar_, have been derived to be ∆S_bar_ = 9.6 J/kgK, 13.5 J/kgK and 13.2 J/kgK for TbMn_2_Ge_2_,Tb_0.9_Y_0.1_Mn_2_Ge_2_ and Tb_0.8_Y_0.2_Mn_2_Ge_2_ respectively. These barocaloric values indicate that these materials can be considered as a potential candidate for mechanocaloric effects over the hydrogen and natural gas liquefaction temperature ranges.

### Heat Capacity

**Figure 7:**
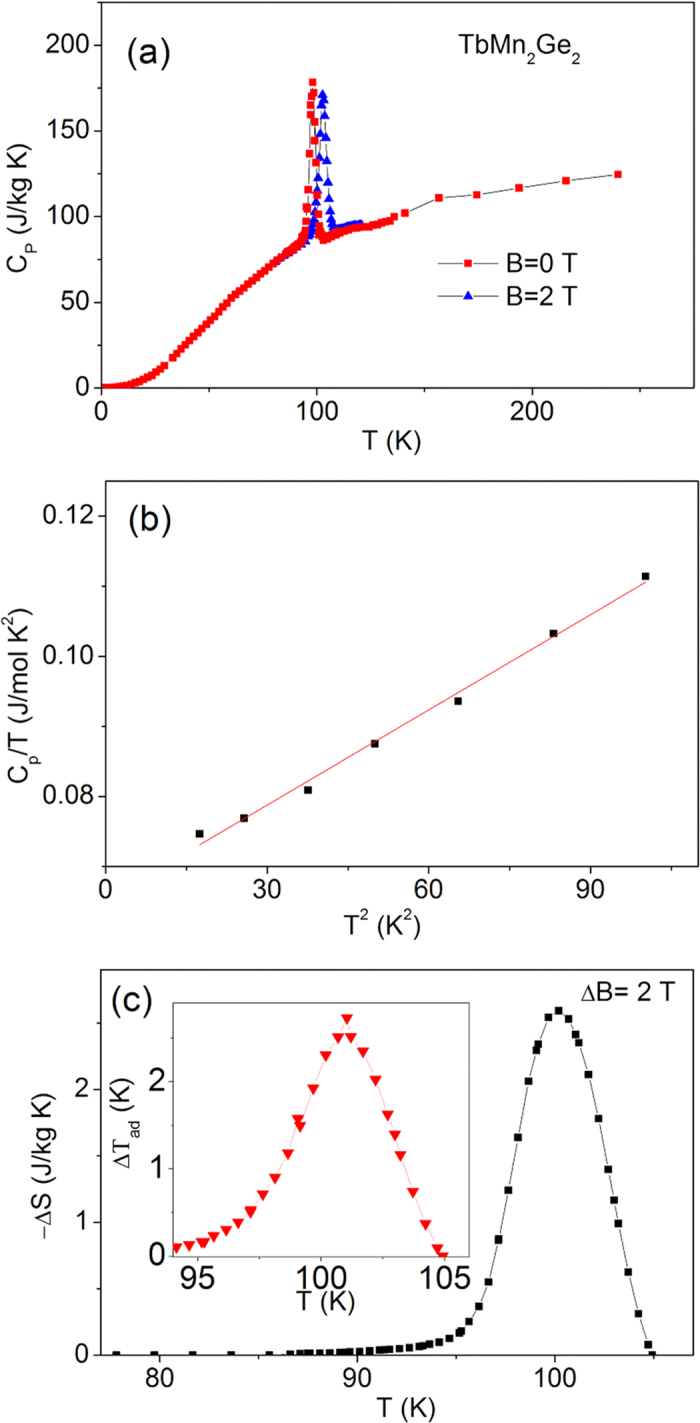
The specific heat capacity relative parameters for TbMn_2_Ge_2_. (**a**) The specific heat capacity C_P_ of TbMn_2_Ge_2_ over the temperature range 2–250 K in zero magnetic field (red solid square) and a field of 2 T (blue solid triangle). (**b**) A graph of C_p_/T versus T^2^ for TbMn_2_Ge_2_ at temperatures below 10 K. (**c**) Magnetic entropy change −ΔS as a function of temperature derived from the specific heat data of Fig. 7(a) for ΔB = 0–2 T. The inset shows the corresponding adiabatic temperature change, ΔT_ad_.

The heat capacity of TbMn_2_Ge_2_ over the temperature range 2–250 K is shown in Fig. 7(a). The sharp peak in the heat capacity near the Curie temperature of TbMn_2_Ge_2_ on both zero magnetic field and a field of 2 T reflects the first order character of the magnetic phase transition. The peak in specific heat shifts from ~98 K to 102.6 K for magnetic fields of 0 T and 2 T respectively; this behaviour corresponds well to the values of the Curie temperature of TbMn_2_Ge_2_ - 97.5 K (B = 0 T) to 103 K (B = 2 T) - obtained for the magnetization measurements (Fig. 3(d)).

The heat capacity C(T) of a metallic magnetic material includes contributions from phonons, electrons and magnons and can be described as follows:


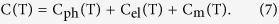


where C_ph_, C_el_ and C_m_ are the lattice, electronic, and magnetic contributions respectively^44^. In the absence of a magnetic phase transition, the heat capacity can be described as:


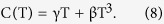


where γ and β are the electronic and phonon heat capacity coefficients, respectively. For the specific heat of TbMn_2_Ge_2_ at low temperatures T ≤ 1 K, well away from the magnetic transition, as shown in Fig. 7(b), a fit to the graph of C_p_/T versus T^2^ leads to γ = (65.2 ± 0.95)mJ/molK^2^, β = (4.53 ± 0.156) × 10^−4^ J/molK^4^. The electronic density of states N(E_F_) at the Fermi surface can be calculated by the formula:^44^


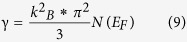


where k_B_ is the Boltzmann constant. For the TbMn_2_Ge_2_ compound, the value of N(E_F_) is derived to be (5.54 ± 0.08) state/eV atom. Likewise, the Debye temperature θ_D_ can also be obtained by:


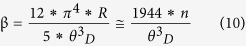


where R is the universal gas constant and the number of atoms n = 5^45^. The Debye temperature for TbMn_2_Ge_2_ was determined as θ_D_ = 278 ± 3 K.

The magnetic entropy change, −ΔS_M_ (T, B) can also be derived from measurements of the in-field heat capacity using the expression thermodynamic relations below:^46^


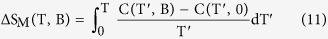


where C(T, B) and C(T, 0) are the values of the heat capacity measured in field B and zero field, respectively. The maximum of magnetic entropy change has been derived to be −ΔS_M = _2.6 J/kgK for TbMn_2_Ge_2_ (field change of ΔB = 2 T), which is smaller than the value (−ΔS_M_ = 5.9 J/kgK) deduced from the isothermal magnetization curves. This behaviour may be due to the fact that a straightforward numerical integration using Maxwell equation based on magnetization curves is not applicable in the phase-separated state as described in ref. 47,48. The corresponding adiabatic temperature change, −ΔT_ad_ (shown as inset of Fig. 7(c) can be evaluated from −ΔS_M_ (T, B) and the heat capacity data.

**Figure 8:**
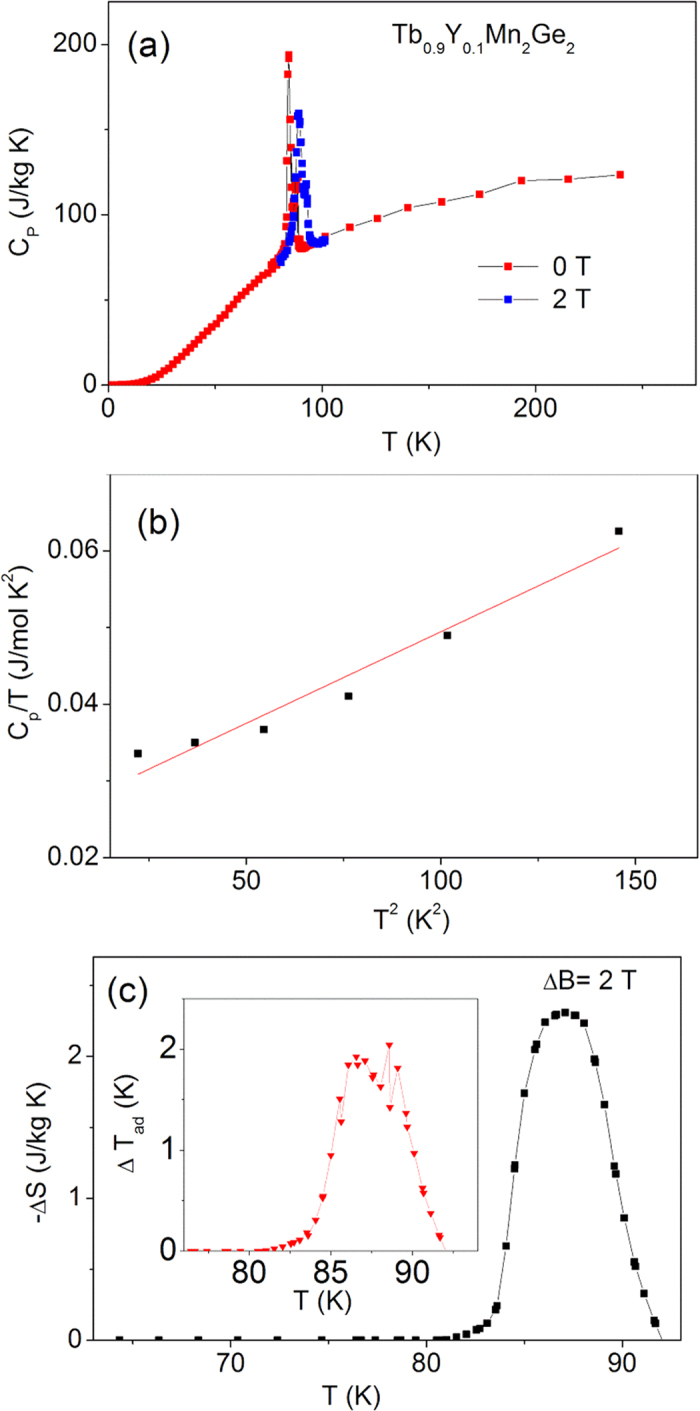
The specific heat capacity relative parameters for Tb_0.9_Y_0.1_Mn_2_Ge_2_. (**a**) The specific heat capacity C_P_ of Tb_0.9_Y_0.1_Mn_2_Ge_2_ over the temperature range 2–250 K in zero magnetic field (red solid square) and a field of 2 T (blue solid triangle). (**b**) A graph of C_p_/T versus T^2^ for Tb_0.9_Y_0.1_Mn_2_Ge_2_ at temperatures below 10 K. (**c**) Magnetic entropy change −ΔS as a function of temperature derived from the specific heat data of Fig. 8a for ΔB = 0–2 T. The inset shows the corresponding adiabatic temperature change, ΔT_ad_.

**Figure 9:**
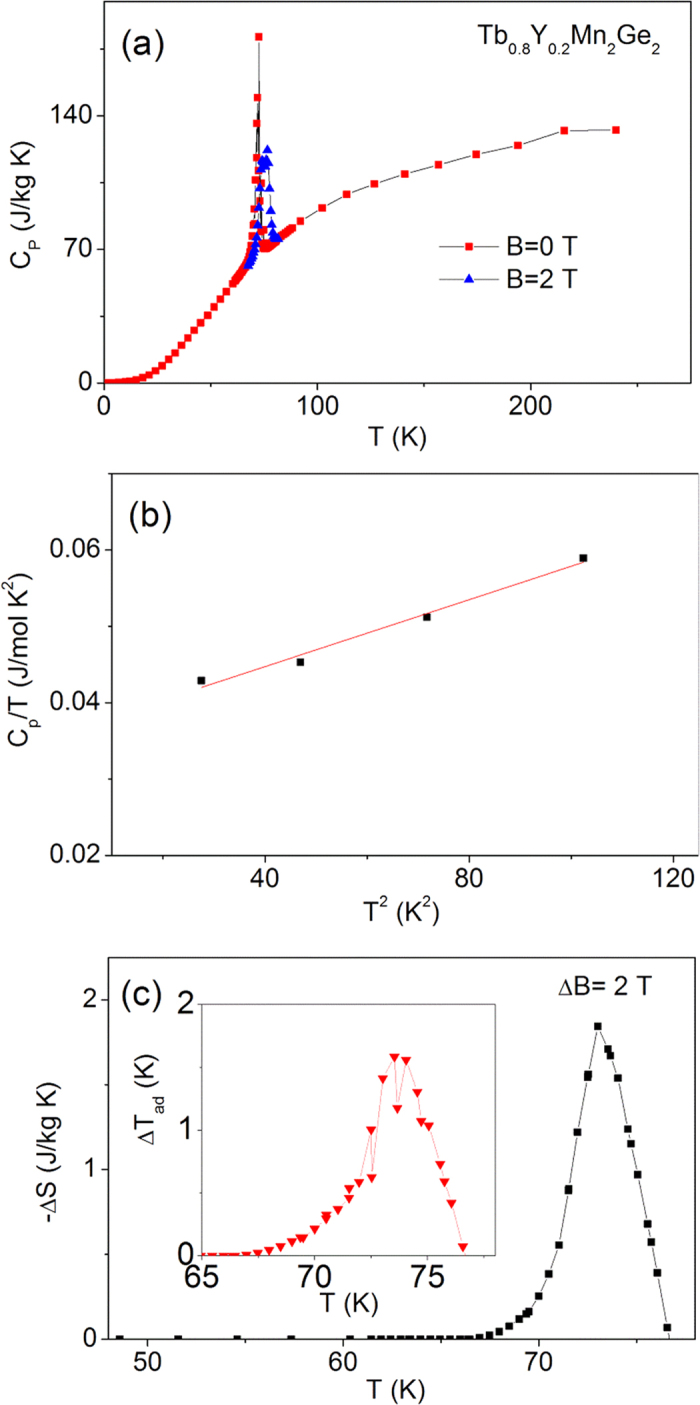
The specific heat capacity relative parameters for Tb_0.8_Y_0.2_Mn_2_Ge_2_. (**a**) The specific heat capacity C_P_ of Tb_0.8_Y_0.2_Mn_2_Ge_2_ over the temperature range 2–250 K in zero magnetic field (red solid square) and a field of 2 T (blue solid triangle). (**b**) A graph of C_p_/T versus T^2^ for Tb_0.8_Y_0.2_Mn_2_Ge_2_ at temperatures below 10 K. (**c**) Magnetic entropy change −ΔS as a function of temperature derived from the specific heat data of Fig. 9(a) for ΔB = 0–2 T. The inset shows the corresponding adiabatic temperature change, ΔT_ad_.

The equivalent heat capacity parameters for Tb_0.9_Y_0.1_Mn_2_Ge_2_ and Tb_0.8_Y_0.2_Mn_2_Ge_2_ are shown in Figs 8 and 9, respectively. The Debye temperatures were found to increase from 281 K for TbMn_2_Ge_2_ to 344 K for Tb_0.9_Y_0.1_Mn_2_Ge_2_ and 354 K for Tb_0.8_Y_0.2_Mn_2_Ge_2_; with the increases understood in terms of the differences in their molecular mass^31^. The adiabatic temperature changes near the Curie temperature are found to decrease from −ΔT_ad_ = 2.6 K for TbMn_2_Ge_2_, to −ΔT_ad_ = 2.3 K for Tb_0.9_Y_0.1_Mn_2_Ge_2_ and 1.8 K for Tb_0.8_Y_0.2_Mn_2_Ge_2_.

The electron density at the Fermi surface is found to decrease from 5.54 state/eV atom for TbMn_2_Ge_2_ to 2.18 state/eV atom and 3.06 state/eV atom for Tb_0.9_Y_0.1_Mn_2_Ge_2_ and Tb_0.8_Y_0.2_Mn_2_Ge_2_ respectively. All the fitting results including electronic heat capacity coefficient γ, phonon heat capacity coefficient β, electronic density of states N(EF) and Debye temperature θ_D_ are summarized in Table 2. The modification of the electron density at the Fermi surface may be related to the difference of electronic configuration of Y and Tb as well as the unit cell size variation. The latter may lead to the variation in the degree of hybridization of Mn 3d states with p states of Ge with decreasing interatomic distances for Y doped samples. Similar behaviour has been found in the La_1−x_Y_x_Mn_2_Si_2_ system where the electron density is derived to be 2.83 states/eV atom for x = 0, 2.51 states/eV atom for x = 0.25, 2.54 states/eV atom for x = 0.3 and 1.47 states/eV atom for x = 1.0^49^. Moreover, it is also noted that the electron density at the Fermi level for TbMn_2_Si_2_^29^ was reported to be 2.38 states/eV atom, which is close to the values reported here for Tb_1−x_Y_x_Mn_2_Ge_2_ samples.

## Conclusions

In conclusion, we have carried out a detailed investigation around the region of the magnetic transitions of compounds in the Tb_1−x_Y_x_Mn_2_Ge_2_ series (x = 0, 0.1, 0.2) by variable temperature x-ray diffraction, heat capacity, differential scanning calorimetry and magnetic measurements. Two magnetic phase transitions occur at T_N_^inter^ and T_C_^inter^ for each of the three samples. The antiferromagnetic transition at T_N_^inter^ is shown to increase slightly with increase in the Y concentration, while the ferromagnetic transition at T_C_^inter^ drops significantly. The mechanism of reduction of T_C_ due to the substitution of Y for Tb has been analysed and chemical pressure is found to play a significant role. Moreover, the entropy change of Tb_0.9_Y_0.1_Mn_2_Ge_2_ is found to exhibit very good magnetocaloric performance around T_C_^inter^ (−ΔS = 11.9 J kg^−1^ K^−1^ and RCP = 102.9 J kg^−1^ for a field change of ΔB = 0–5 T) with a small hysteresis loss of 5.36 J/kg. This behaviour reflects the potential suitability of Tb_0.9_Y_0.1_Mn_2_Ge_2_ for operation as a magnetic refrigerant below the nature gas liquefaction temperature. The Debye temperature and the density of states N(E_F_) at the Fermi level have been determined and analyzed from the heat capacity.

## Additional Information

**How to cite this article:** Fang, C. *et al*. New insight into magneto-structural phase transitions in layered TbMn_2_Ge_2_-based compounds. *Sci. Rep.* **7**, 45814; doi: 10.1038/srep45814 (2017).

**Publisher's note:** Springer Nature remains neutral with regard to jurisdictional claims in published maps and institutional affiliations.

## Figures and Tables

**Table 1:** Experimental data for the three Tb_1−x_Y_x_Mn_2_Ge_2_ samples (x = 0, 0.1 and 0.2).

x	0	0.1	0.2
Δp (Gpa)	0	0.012	0.053
V (Å^3^)(300 K)	173.66(5)	173.63	173.53
V (Å^3^)	0.40	0.52	0.54
Δμ (Am^2^/kg) (B = 1 T)	14.0(5)	28.2	22.7
experimental T_c_ (K) (FC)	93.0(5)	81.8	66.0
ΔT_c_^chemical^ (K)	0	1.94	8.70
ΔT_c_^total^ (K)	0	11.2	27.0
ΔT_c_^chemical^/ΔT_c_^total^	0	17.3%	32.2%
dT_c_/dB (cooling) (K/T) (FC)	1.06 ± 0.04	1.54 ± 0.05	1.29 ± 0.09
dT_c_/dp (K/kbar) (FC)	−3.03	−2.84	−3.07

Y composition x, chemical pressure Δp, unit cell volume at 300 K, the change in unit cell volume Δ*V*_*m*_ and the change in moment Δμ on magnetic field 1 T during the structural transition, value of T_c_ during the FC process, the value of dT_c_/dB, the total difference value of Curie temperature ΔT_c_^total^ between Tb_1−x_Y_x_Mn_2_Ge_2_ (x = 0.1 and 0.2) and TbMn_2_Ge_2_, the derived values of ΔT_c_^chemical^ (caused by chemical pressure) and dT_c_/dp of Tb_1−x_Y_x_Mn_2_Ge_2_ (x = 0, 0.1 and 0.2). The errors are shown for the TbMn_2_Ge_2_ data as an example.

**Table 2:** Calculated heat capacity parameters for Tb_1−x_Y_x_Mn_2_Ge_2_ (x = 0, 0.1, 0.2).

x	γ (mJ/molK^2^)	β (J/molK^4^)	N(E_F_) (state/eV atom)	θ_*D*_(K)
0	65.2 ± 0.9	(4.53 ± 0.16) × 10^−4^	5.54 ± 0.08	278 ± 3
0.1	25.6 ± 2.0	(2.39 ± 0.24) × 10^−4^	2.18 ± 0.17	345 ± 12
0.2	36.0 ± 1.3	(2.19 ± 0.19) × 10^−4^	3.06 ± 0.11	355 ± 11

Electronic heat capacity coefficient γ, phonon heat capacity coefficient β, electronic density of states N(E_F_) and Debye temperature θ_*D*_.
